# Leptin, resistin and visfatin: the missing link between endocrine metabolic disorders and immunity

**DOI:** 10.1186/2047-783X-18-12

**Published:** 2013-05-01

**Authors:** Ebtesam A AL-Suhaimi, Adeeb Shehzad

**Affiliations:** 1 Department of Biology, Sciences College, University of Dammam, Dammam, Saudi Arabia; 2 School of Life Sciences, College of Natural Sciences, Kyungpook National University, Daegu 702-701, South Korea

**Keywords:** Adipocytokines, Obesity, Diabetes, Endocrine, Immunity

## Abstract

Adipose tissue is still regarded as a principle site for lipid storage and mobilizing tissue with an important role in the control of energy homeostasis. Additionally, adipose tissue-secreted hormones such as leptin, visfatin, resistin, apelin, omentin, sex steroids, and various growth factors are now regarded as a functional part of the endocrine system. These hormones also play an important role in the immune system. Several *in vitro* and *in vivo* studies have suggested the complex role of adipocyte-derived hormones in immune system and inflammation. Adipokines mediate beneficial and detrimental effects in immunity and inflammation. Many of these adipocytokines have a physiological role in metabolism. The uncontrolled secretions of several adipocytokines were associated with the stimulation of inflammatory processes leading to metabolic disorders including obesity, atherosclerosis, insulin resistance and type 2 diabetes. Obesity leads to the dysfunction of adipocytes andcorrelated with the imbalance of adipokines levels. In obese and diabetic conditions, leptin deficiency inhibited the Jak/Stat3/PI3K and insulin pathways. In this review, ample evidence exists to support the recognition of the adipocyte’s role in various tissues and pathologies. New integral insights may add dimensions to translate any potential agents into the future clinical armamentarium of chronic endocrine metabolic and inflammatory diseases. Functional balance of both adipocytes and immune cells is important to exert their effects on endocrine metabolic disorders; furthermore, adipose tissue should be renamed not only as a functional part of the endocrine system but also as a new part of the immune system.

## Review

## Introduction

Adipose tissue is a complex network of endocrine organs that has been divided into white adipose tissue (WAT) and brown adipose tissue (BAT). WAT is mainly responsible for the insulation and mechanical support along with the energy storage function in the body, while BAT specializes in thermogenesis and lipid oxidation [1]. Activation of BAT also regulates channel efficiency to undertake triglyceride-rich lipoprotein (TRL) clearance, as well as to prevent excess accumulation of lipids in the blood [1].

The most abundant depots are visceral and subcutaneous adipose tissues, which produce unique profiles of adipokines [2]. These molecules are orchestrated by a multifarious network belonging to different functional categories such as immunity (complement factors, haptoglobulin), endocrine function (leptin, adiponectin, visfatin, resistin, apelin, omentin, sex steroids, various growth factors), metabolic function (fatty acids, adiponectin, resistin, vaspin), cardiovascular function (angiotensinogen), fatty acid, and prostaglandins [3-7].

Adipose tissue secretes many biologically active adipokines with diverse functions [3]. Adipokines are pharmacologically active, low molecular weight proteins that exert pleiotropic functions through several metabolic pathways [8]. Around the turn of the 20^th^ century several molecular mechanisms shed light on the importance of adipokines in the human system. To date, more than 20 different hormones (both orexigenic and anorexigenic) have been identified. Adipokines are important due to their crucial mediator role and active participation in metabolic functions. These hormones also easily cross the blood-brain barrier, reach the main site of action located in the hypothalamic region, exert their actions and mediate the balance of satiety and hunger. The adipokines have a central role in the control of energy metabolism, communicating the nutrient status of the organism including energy intake and expenditure as well as insulin sensitivity [9].

Adipokines have several mediators such as adiponectin, pre-B cell colony-enhancing factor (PBEF) visfatin, leptin, resistin and retinol-binding protein-4. Adiponectin is an intriguing adipokine with its serum level inversely correlated with fatness. It is related to the enhancement of insulin sensitivity, anti-inflammatory effects, anti-atherogenic actions, and regulation of metabolic homeostasis [10]. Recent investigations have also emphasized the importance of adipocytokines such as interleukin-6 (IL-6), tumor necrosis factor α (TNFα), plasminogen activator inhibitor-1, or a chemokine, and monocyte chemoattractant protein-1 (MCP-1). Nuclear factor-kappa B (NF-κB) is a transcription factor that has the potential to mediate immunity, stress, apoptosis, cytokines expression, inducible nitric oxide synthase (iNOS), cyclo-oxygenase 2 (COX-2), cell growth factors, and development, as well as the potential to play an important role in central nervous system and cell signaling. It is also known that NF-κB induces overexpression of transcriptional systems, which activate inflammation and develop cancer. NF-κB pathway inhibitors may be a useful therapeutic strategy to treat inflammation and cancer. Several studies have shown that lipid accumulation in the liver leads to hepatic inflammation through NF-κB activation and downstream cytokine production, which leads to insulin resistance hepatically, as well as systemically [11,12]. Some of the adipokines hormones such as leptin, adiponectin, resistin, and ghrelin play a role in the regulation of glucose metabolism and are involved in the development of obesity, diabetes, inflammation, auto-immunity and metabolic syndromes [13].

The current review summarizes the recent knowledge regarding the malfunction of adipokines such as leptin, resistin, and visfatin in the initiation and progression of many metabolic diseases including obesity, diabetes and immunity.

### Physiological and pro-inflammatory roles of adipokines

The physiological, metabolic and pro-inflammatory role of different adipokines, such as, leptin, resistin, visfatin will be discussed individually and according to the role each plays in diabetes, obesity, and immunity.

#### Leptin

The discovery of leptin has led to a new era in nutrition biology. Leptin was discovered in mice in 1994 by Jeffrey M. Friedman. Leptin is derived from the Greek word leptos, which means ‘thin’. The serum concentration of leptin is predominantly defined by body fat mass [14]. Leptin was the first identified adipocytokine, its primary structure is composed of 167 amino acids, and it is primarily expressed in adipose tissue. Leptin regulates energy homeostasis and interferes with several neuroendocrine and immune functions [15]. A higher amount of leptin is secreted by subcutaneous adipocytes than by the visceral adipocytes. Its presence has also been detected in many other tissues, including the placenta, mammary glands, breast milk, testes, ovaries, endometrium, stomach, hypothalamus, and pituitary gland [16]. Leptin is generally synthesized and secreted by gastric chief cells in the stomach [17]. Leptin circulating levels are directly proportional to the body fat. These levels range from 5 to 10 ng/ml in healthy individuals to 40 to 100 ng/ml in obese individuals [18]. A transient increase occurs during a meal, whereas leptin levels decrease with fasting, evoking a profound changes in energy balance and hormone levels [4,19,20].

The adipose tissue secretes specific proteins including leptin into the blood stream, which controls body weight by regulating metabolic behavior. It has a fundamental role in the control of appetite and also in regulating energy expenditures [21]. Leptin exerts pleiotropic effects by binding and activating specific leptin receptors (obR) in the hypothalamus and other organs. It has direct and indirect effects in metabolically active tissues and regulates several neuroendocrine axes [22,23]. So far, six different isoforms of leptin have been identified with diverse biological actions that range from affecting blood pressure to affecting immune functions [24,25]. Leptin receptors such as ObRa and ObRb are present in the brain and regulate metabolic behaviors [26]. Among leptin receptors, mRNA expression of the long form, ObRb, has been detected in the arcuate (ARC), dorsomedial (DMH), ventromedial (VMH), and ventral premamillary nuclei (PMV); moderate expression has been found in the periventricular hypothalamic nucleus, in the lateral hypothalamic area (LHA), and at lower levels of expression in the paraventricular nucleus (PVH) [27]. Early research was focused on leptin and its receptors in the hypothalamus region and leptin was believed to be an important regulatory hormone for signaling body fat status. However, it has become apparent that leptin receptors are expressed in many normal cell types throughout the body as well as in malignant cells. It is noted that the addition of leptin to cells in culture was found to promote proliferation and to inhibit apoptosis [27-29]. Leptin has been implicated as a growth factor for its ability to stimulate angiogenesis in metastatic breast cancer hypoxic conditions [30]. Angiogenesis is also a crucial factor in determining obesity and its related complications [31].

#### Resistin

Resistin is another unique adipocyte-derived signaling cysteine-rich molecule made up of 114 amino acids, and was first identified in obese mice. The resistin-like molecule (*RELM*) gene family is an N-terminal signal peptide. Resistin (FIZZ3), known as the resistin-like molecules RELM; RELM, α/FIZZ 1, and RELMβ/FIZZ 2, is involved in various inflammatory processes [32-34]. In addition, human resistin has also been detected in tissues like placenta, skeletal muscle, small intestine, spleen, stomach, thymus, thyroid gland and uterus [35-37].

Resistin expression was greater in white adipose tissue than in brown adipose tissue [35]. The resistin in mice is expressed in white adipose tissue, whereas human resistin is expressed in adipose tissue at a lower level [38]. However, resistin is predominantly expressed in macrophages in humans. Resistin is thus named because of its resistance to the action of insulin. It has been observed that circulating resistin levels are increased in obese humans. It is considered a pro-inflammatory molecule, which also plays an important role in the pathogenesis of diabetes and its complications. The release of resistin is often stimulated by the inflammatory process, IL-6, hyperglycemia and hormones such as growth hormone and gonadal hormones [39].

#### Visfatin

Visfatin, also known as pre-B cell colony-enhancing factor (PBEF), is a highly conserved, 52-kDa protein found in living species from bacteria to humans [40]. Visfatin is also called NAMPT because of its significant sequence and functional homology with nicotinamide phosphoribosyltransferase (NAm-PRTase), an enzyme involved in nicotinamide adeninedinucleotide (NAD) biosynthesis from nicotinamide [41]. It is produced by the visceral adipose tissue. The expression of visfatin is increased in individuals with abdominal obesity and type 2 diabetes [38].

### Leptin, resistin and visfatin and their relation to obesity

#### Leptin and obesity

In recent years a remarkable progress has been made in the understanding of obesity pathophysiology. The more recent findings have corroborated that leptin may signify a link between obesity and metabolic disorders [41]. In normal mice, the leptin interacts with the products of leptine receptor (Lepr) locus [42] in the choroid plexus [43]. It is postulated that leptin deficiency occurs in the genetically obese Lep^ob^ mouse [44].

Genetically obese mice strains were discovered because of a mutation in the leptin receptor [42]. Lep^ob^ mouse have elevated levels of leptin in serum, which is the hallmark of the relationship between leptin and its positive association with body weight, body mass index (BMI), and fat mass [45-47]. Diabetes (db/db) mice have a deletion in the long isoform of the leptin receptor; therefore, it is resistant to leptin [48]. Leptin circulates proportionally to adipocytes, regulating food intake and energy expenditure through the expression of ObRb receptors in the central nervous system (CNS) [49]. In addition, leptin binds to the neuropeptide neuron in the mediobasal hypothalamus and signals the brain regarding the adipose stores, producing appropriate conditions of satiety through neurotransmitters and other related hormones. Experimental studies have implicated leptin as a crucial controller of body weight through central and peripheral pathways [50] because circulating leptin conveys information to the hypothalamus regarding the amount of energy stored in adipose tissue; suppresses the appetite, affects energy expenditure [51], affects weight reduction; and significantly increases with the circulating levels of adiponectin. However, the two hormones perform complementary actions, and may have synergistic effects [52].

**Figure 1:**
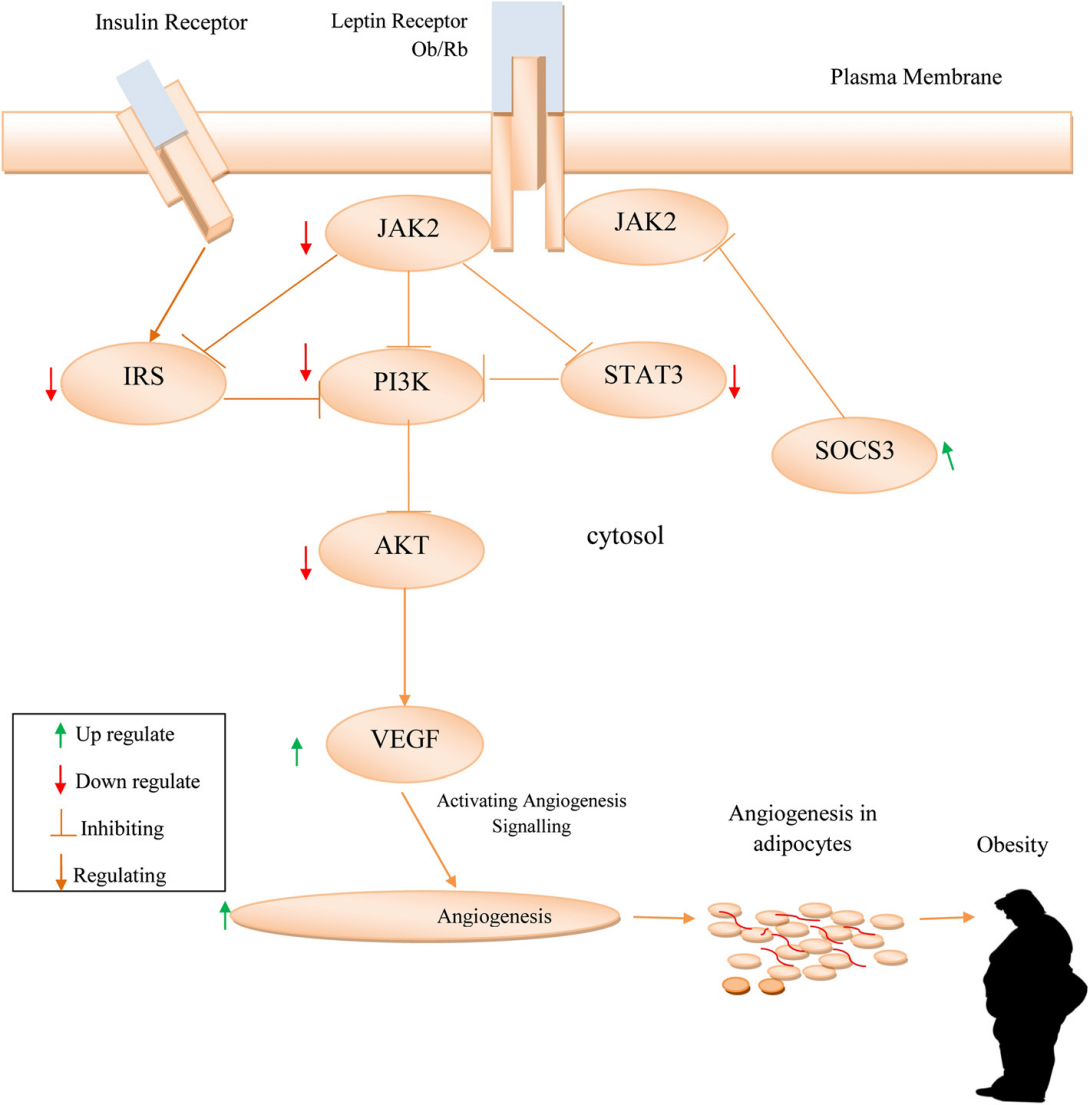
Obstruction of Jak/Stat3/PI3K-dependent pathways stimulated VEGF to stimulate angiogenesis in adipocytes thereby leading to obesity.

The long isoform of the leptin receptor (Ob Rb) is a vital activator of janus kinase signal transduction and translation/signal transducer and activator of transcription (JAK/ STAT) pathway [49]. It has been reported that the JAK/ STAT pathway is responsible for leptin regulation of energy homeostasis [53,54]. Suppressor of cytokine signaling 3 (SOCS-3) protein is a negative feedback regulator of leptin signaling involved in leptin resistance. The potential mechanism contributing to leptin resistance occurs through upregulation of SOCS-3 expression by inhibiting the signal transduction stage of leptin receptor [55,56]. The suppressor of cytokine signaling (SOCS) protein inhibits the JAK-STAT pathway and leads to leptin resistance and obesity [57]. In the hypothalamus the phosphatidylinositol 3-kinase (PI3K) pathway interaction with the JAK2/STAT3 cascade plays a significant role in the signal transducing leptin action [58]. In obese conditions the leptin resistance downregulates the Src/PI3K/Akt pathway [59]. Obstruction of the Jak/Stat3/PI3K-dependent pathways upregulates the effects of vascular endothelial growth factor (VEGF) to enhance angiogenesis in adipocytes [60-62]. In mice, such a disrupted JAK/STAT pathway results in an increased food intake and accumulation of adipose tissue [53]. Buettner *et al*. has reported that the restoration of STAT3 signaling is an effective therapy for leptin defective diseases [63]. In obese condition, leptin resistance is accompanied by hyperinsulinemia and insulin resistance [64] (Figure 1).

#### Resistin and obesity

Serum resistin was positively correlated with changes of BMI and body adipose mass. Circulating resistin levels increase with age, probably reflecting the increase in the body fat content [65]. With human obesity elevated serum resistin levels were observed when compared with humans in lean condition [66]. Resistin is involved in the proliferation of adiposities and angiogenesis [66-69]. Obesity is associated with abnormally elevated JNK activity, predominantly provided by JNK1. It is a vital component of the obesity-induced insulin resistance pathway *in vivo*[70]. Scientists have suggested that resistin is a hormone that links obesity to diabetes. Experiments in humans have shown no differences in resistin expression among normal, insulin-resistant, and type 2 diabetic samples. However, some recent genetic studies have demonstrated an association between resistin and insulin resistance and obesity [71]. Resistin has been shown to antagonize insulin action. Resistin levels are increased in diet-induced obesity as well as in genetic models of obesity and insulin resistance. Also, resistin gene expression is markedly down-regulated by treatment with anti-diabetic drugs called thiazolidinediones that improve target-tissue sensitivity to insulin. It was found that human abdominal adipose tissue has a higher amount of resistin mRNA than other fat depots [72]. Abdominal adipose tissue is thought to be a main risk factor for insulin resistance. It has also been reported that resistin is expressed in the hypothalamus and is able to activate hypothalamic neurons [73,74]. Another study showed that central administration of resistin resulted in increased number of cells expressing Fos (c-Fos is a protein encoded by the FOS gene) in the arcuate nucleus and promoted short-term satiety in rats [75].

#### Visfatin and obesity

Several studies have observed no difference in visfatin mRNA expression in visceral and subcutaneous adipose tissue in humans [75]. However, other studies confirmed an increased level of circulating visfatin whereas results from other studies were contradictory in that they showed lower levels of plasma visfatin in obese subjects [76-80]. It was also reported that overnutrition downregulated circulating visfatin concentrations in humans [81]. The controversial findings on visfatin levels as a result of obesity and metabolic syndrome, suggest that an increased [82], a decreased [83,84], or unchanged level of visfatin-induced endothelial angiogenesis occurs through mediating VEGF, MMP, MAPK and PI3K/Akt signaling pathways [85].

### The relation of leptin, resistin and visfatin with diabetes

#### Leptin and diabetes

Leptin deficiency not only leads to obesity, but also to diabetes and to reproductive dysfunction. An interruption of hepatic metabolism of glucose, fatty acids, and lipoproteins in the leptin-deficient obese (Lep^ob/ob^) mouse leads to hyperglycemia, steatosis, and hypercholesterolemia [86]. Leptin and insulin levels are directly interconnected with body weight and adipose tissue [87]. Leptin has impressive effects on the energy homeostasis, including regulation of insulin secretion by pancreatic β cells [88]. In the brain, insulin and leptin act to inhibit the appetite [89]. Leptin may also directly affect the metabolism and function of peripheral tissues. Leptin has been implicated in causing peripheral insulin resistance by attenuating insulin action, and perhaps insulin signaling, in various insulin-responsive cell types. Additionally, various researchers have demonstrated a significant relationship between leptin and insulin [90].

Insulin and leptin influence the glucose metabolism by acting at a peripheral and central level [91]. They act as adiposity signals and play a pivotal role in the central regulation of energy homeostasis [92]. Both hormones circulate at proportional levels to body fat and regulate food intake and energy expenditure by interacting with their respective receptors [93]. Levi *et al*. reported that leptin administration was able to increase plasma IGFBP-2 levels and improve glucose homeostasis in both ob/ob mouse models [94].

**Figure 2:**
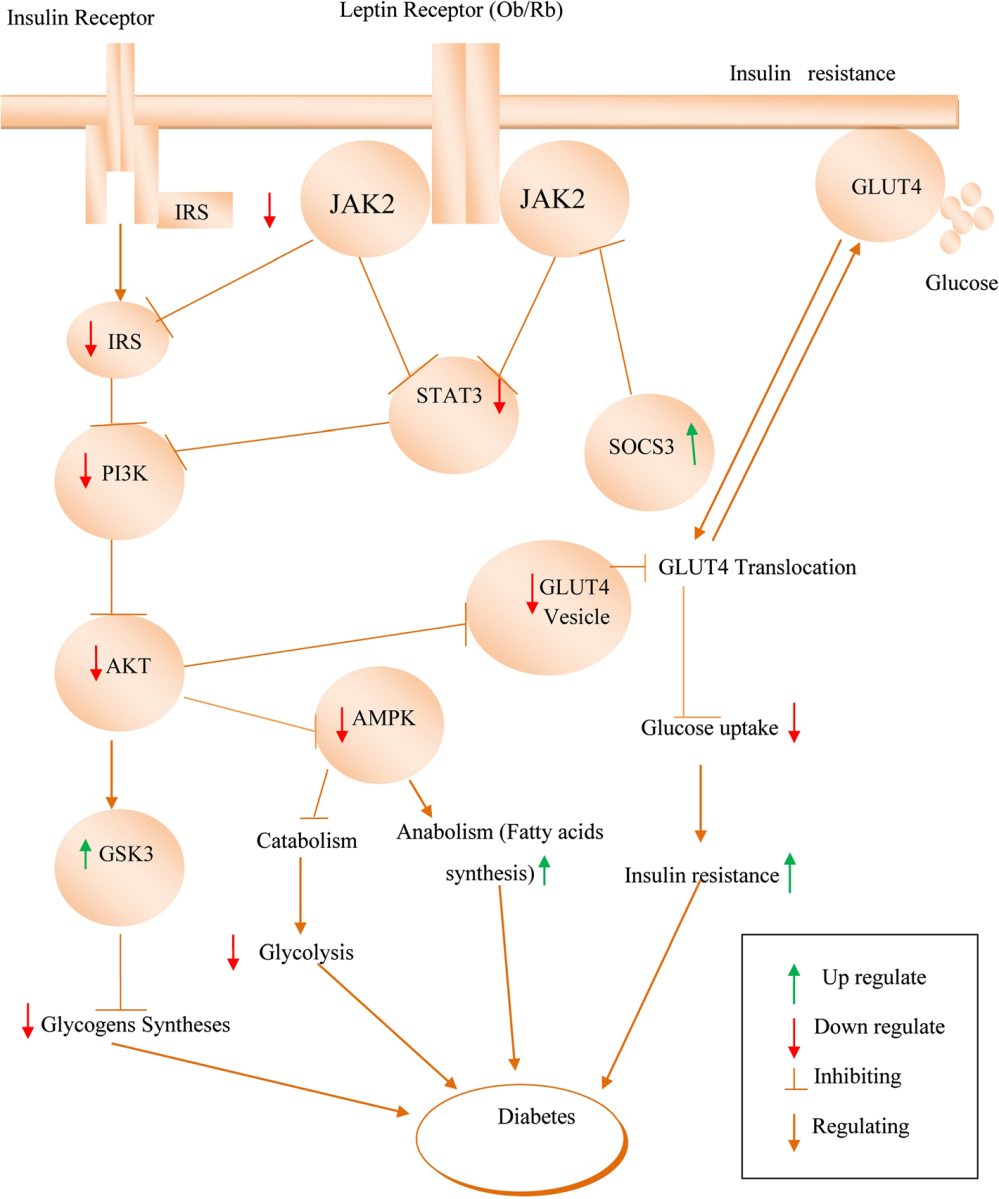
Leptin deficiency disturbs the insulin signaling pathway JAK2/IRS/PI3K/AKT/AMPK and triggers diabetes.

Early reports showed that insulin and leptin play a significant role in diabetic condition by activating the insulin receptor substrate (IRS)-PI3K pathway (Figure 2). Schultze *et al*. reported that the development of insulin resistance and T2DM occurs with stimulated PI3K and downregulation of IRS proteins [95]. Insulin acts by modulating glucose metabolism via the STAT3 permissive effect. Initiation of STAT3 is necessary for the P13K activation [96]. Akt is a critical central mediator that acts along with PI3K for insulin signaling [97]. In addition, PI3K and Akt involve in insulin-stimulated translocation of the glucose transporter type 4 (GLUT4) [98,99].

Under normal circumstances, GLUT4 is responsible for insulin-stimulated glucose uptake from circulation into skeletal muscle [100]. It is reported that GLUT4 levels are reduced in the muscle of Type 2 diabetic condition [101]. Glucose uptake is regulated by GLUT4 and retained by activated PI3K [102]. Glucose uptake into the cells is stimulated by insulin and leptin activating the JAK2/IRS/PI3K/AKT signaling pathway [103] and triggering the translocation of the GLUT4 from the cytosol to the cell surface [104] Downregulation of PI3K and AKT indicates an insufficiency of insulin to maintain the normal signaling [105] and downregulates the intake of glucose through GLUT4 in the muscles, which results in hyperglycemia [106]. Glycogen synthase kinase-3 (GSK-3) is a serine/threonine protein kinase that has multiple negative impacts in insulin-mediated metabolic diseases [107,108]. GSK-3 adversely affects the insulin role by hindering activation of glycogen synthase, leading to the accrual of glycogen in the muscle [109].

AMP-activated protein kinase (AMPK) is an imperative enzyme in leptin signaling related to PI3K pathway [110]. Both PI3K/AKT and AMPK are involved in regulating glucose homeostasis [95]. In normal condition, AMPK stimulates catabolism and impedes anabolic pathways. Leptin deficiencies decrease the AMPK activity in the liver but increase its activity in the hypothalamus of the diabetic rat, thereby resulting in diabetic hyperphagia [111]. In diabetic ob/ob mice, the insulin signaling pathways were deregulated, which are found to be reinstated by systematic leptin treatment [112].

Apart from the diabetes, leptin also affects a variety of other physiological functions, including fertility, bone metabolism, and immune responses [113].

#### Resistin and diabetes

The role of adipocyte hormones in modulating insulin sensitivity and glucose tolerance are of common interest and importance in studies of type 2 diabetes mellitus. Recently, resistin has been proposed to play an important role in the pathogenesis of obesity-related insulin resistance [114]. Previous studies have postulated the controversial role of resistin in obesity and insulin resistance. Some studies have shown a positive correlation with body fat mass and insulin resistance [114,115], whereas others have found no correlation with body mass index (BMI) or insulin sensitivity [116,117]. Resistin is expressed in pre-adipocytes and adipocytes, which may promotes resistin elevation in the adipose tissue of obese human [117,118]. Resistin is produced primarily by adipose tissue and may act at sites distant from where it is produced. In humans, insulin resistances are positively correlated with expression of resistin [119]. In diabetic patients, serum levels of resistin are nearly 50 ng/ml. Resistin increases insulin resistance with respect to carbohydrate metabolism [120].

An elevated expression of resistin in circulation leads to glucose intolerance, hyperinsulinemia related with impaired insulin signaling in skeletal muscle, liver, and adipose tissue. The important role of AMPK in the liver is to stimulate the fatty acid oxidation, thwart cholesterol synthesis and intonate insulin secretion by pancreatic β cells. Resistin inhibits the phosphorylation of the hepatic AMPK pathway that downregulates β oxidation to lipid accumulations [121]. Subsequently, resistin stimulates SOCS-3 in mice adipose [122]. The stimulated SOCS-3 inhibits the insulin signaling pathway in tissues. Moreover, resistin affects glycogen metabolism, leading to type 2 diabetes [123].

An elevated level of circulating resistin was detected in obesity and diabetes. This discovery suggests that deregulation of resistin induces insulin resistance in genetic models (ob/ob and db/db) and in a diet-induced model of diabetes and obesity. Studies have shown that loss of resistin in obesity decreases the blood glucose levels and improves insulin sensitivity [124]. Paradoxically, db/db mice were not found with elevated levels of resistin when compared with the control group, nor has variation in resistin mRNA levels been reported in ob/ob mice. This means that some oversecretion of resistin from adipocytes may decrease the total number of resistin per cells, highlighting the tissue and cell-specific effect of resistin [123,125]. In humans, resistin is primarily released by monocytes/macrophages, suggesting that soluble levels may be associated with macrophage activation. Here, systemic and monocyte-released resistin levels were found to be similar in type 2 diabetic patients, overweight controls and normal-weight controls [126]. The aforementioned results strongly support the role of human resistin in the development of insulin resistance and inflammation. Thus, human resistin may be linked from insulin resistance to inflammatory diseases such as obesity, type 2 diabetes, and atherosclerosis [127].

#### Visfatin and diabetes

In adipokines, resistin is considered important for their pro-inflammatory effects. Alternatively, visfatin is known for insulin-mimetic/sensitizing effects. It is overexpressed in the route of adipocyte differentiation. Studies have shown that the synthesis and secretion of visfatin is modulated by glucocorticoids, TNFα, IL-6, and growth factors. This means that visfatin is upregulated in the course of pro-inflammatory cytokines and under inflammatory conditions. Also, the findings of McGee *et al*. have suggested that visfatin may represent a pro-inflammatory cytokine that is influenced by insulin/insulin sensitivity via the NF-κB and JNK pathways [128].

The biological role of visfatin is not entirely understood, but several studies indicated glucose lowering and insulin-mimicking/-sensitizing effects of visfatin. Heterozygous mice with a target mutation in the visfatin gene had modestly higher levels of plasma glucose impaired glucose tolerance and reduced glucose-stimulated insulin secretion relative to control mice [128]. But there was no significant correlation of plasma visfatin levels and parameters of insulin sensitivity, including fasting insulin, fasting plasma glucose concentrations, and the glucose infusion rate during the steady state of a euglycemic hyperinsulinemic clamp independent of percent body fat. There are also conflicting data on visfatin circulating levels in obese humans.

### The relation of leptin, resistin and visfatin with immunity

#### Leptin and immunity

Leptin’s role in immune responses has been recently reviewed. It modulates the monocytes/macrophages, neutrophils, basophils, eosinophils, natural killer and dendritic cells. Leptin modifies T-cell balance, induces T-cell activation, and changes the pattern of T-cell cytokine production by driving T-cell differentiation towards a T-helper1(TH1) response. This led to studies of the pro-inflammatory role of leptin in several animal models of autoimmune /inflammatory conditions. Studies have shown that leptin modulation in the immune system is mediated at development, proliferation, maturation and production levels [129]. However, the functional role of leptin is abrogated in the immune cells through the modulation of multiple signaling pathways, including STAT-3, PI3K, and P38 mitogen-activated protein kinase (MAPK) [130]. In fact, leptin and leptin receptors have pleiotropic effects in immune cells, promoting T-helper 1 responses, natural killer cell cytotoxicity, and production of inflammatory cytokines such as C-reactive protein (CRP), IL-6, TNFα, and serum amyloid A [131]. It is believed that leptin is a pro-inflammatory adipokine that induces T helper 1 cells and may contribute to the development and progression of autoimmune responses [132]. Leptin receptor is also upregulated by aforementioned pro-inflammatory signals [133]. Results showed that peripheral rather than central leptin treatment was able to significantly increase numbers of granulocytes, Nutral Killer cells (NK) cells and monocytes [134]. Furthermore, it characterized NK cell differentiation and maturation in the bone marrow of leptin-receptor deficient db/db mice at a prediabetic stage [135]. Leptin signaling regulates NK cell development via enhancing the survival of immature NK cells in mouse bone marrow. A lot of compelling evidence has shown that leptin is the connection between nutritional status and immune competence. Leptin has been shown to regulate the immune responses in innate and adaptive response in normal and pathological conditions [134]. A study of adult leptin receptor-defective (db/db) mice demonstrated a significant reduction of both NK cell numbers and NK cytotoxic capacity compared with wild-type mice. Treatment of NK (CD56+) cells with leptin enhanced CD69 expression and stimulated splenic NK cytotoxicity in wild-type mice, but not in db/db mice. Furthermore, the decreased number of NK cells in the db/db mice has confirmed the role of leptin in the maturation of NK cells [136]. On the other hand, leptin-activating pro-inflammatory cytokines such as IL-6 and TNF-α protect Ob/ob mice from T cell hepatotoxicity [136]. Leptin mediate the inflammatory infiltrate and could act as a monocyte/ macrophage chemoattractant, inducing *in vitro* maximal chemotactic responses at 1 ng/mL [137] and inducing tissue-factor expression in human peripheral blood mononuclear cells [138]. Epidemiological observations indicated that reduced leptin production is closely associated with increased infection, which is the major cause of inflammatory or immunodeficiency diseases. Indeed, starvation or malnutrition is one of the human health concerns that increased the vulnerability to infections [132]. Conversely, immune-mediated disorders such as autoimmune diseases are associated with increased secretion of leptin and production of pro-inflammatory pathogenic cytokines. Thus, leptin is a mediator of the inflammatory response [139]. Furthermore, leptin treatment directly affects the lymphocytes and increases the differentiation and proliferation of CD4+ T cells [140].

Leptin appears to modulate TH cells and leading to stimulate TH1 production of some cytokines: interleukin 2 IL-2, interferon gamma (IFN-γ), TNF-α and IL-18, and inhibits the production of TH2 cytokines: IL-4, IL-5 and IL-10 [140], But T lymphocytes from db/db mice do not show the same result, which supports the idea of the direct effect involving the expression of leptin receptors on the T lymphocytes [141]. Currently, leptin replacement therapy enhanced T-cell responsiveness and inducing T-helper 1 cells and may contribute to the development and progression of autoimmune responses [132]. Modifications in the relative proportions of the lymphocyte classes depend on the degree of obesity, or on leptin concentration, or even fat depot anatomical location. It is suggested that alterations in the number and composition of lymphocytes precede an increase in macrophages and enhance the inflammatory state of WAT found in obesity. Lymphocytes express receptors to adipokines while several pro-inflammatory chemokines are produced in WAT, rendering intricate crosstalk between fat and immune cells [142].

#### Resistin and immunity

More recently, it was found that high resistin levels predicted favorable anti-inflammatory effects of inhaled glucocorticoids suggesting that resistin may be a marker of a steroid-sensitive phenotype in asthma [143]. Resistin might contribute to the inflammatory conditions by mediating enhanced activation of cytokines (IL-6, TNFα) and NF-κB [124]. Analyses of resistin gene expression across a wide array of human tissues revealed that peripheral blood mononuclear cells (PBMCs), macrophages and bone marrow cells are the major sources of human resistin [36,144]. Several studies demonstrated that inflammatory stimuli mediate resistin production. In human PBMCs, pro-inflammatory cytokines such as IL-1, IL-6 and TNF- α, as well as Lipopolysaccharides (LPS), have strongly induced resistin mRNA expression [144]. Resistin significantly enhanced the hepatic inflammation and necrosis in LPS-induced liver damage in mice. This effect of resistin was presumably mediated via activation mechanisms involving the coagulation cascade and fibrin accumulation [145]. Resistin is likely to play an important role in chronic inflammatory and autoimmune diseases [146]. An increased level of circulating resistin was also observed in patients with chronic pancreatitis, suggesting its impact on pancreatic fibrosis development [147]. However, circulating resistin levels were clearly associated with general inflammation, renal disease, treatment with glucocorticoids, and bone loss in systemic lupus erythematosus patients [148].

#### Visfatin and immunity

Visfatin is not only an adipocyte-specific protein; its expression gene was originally found in human peripheral blood lymphocytes. It increases the effect of IL-7 and stem-cell factor on pre-B cell colony formation [149]. Visfatin appears to be an important mediator of inflammation. It is demonstrated that recombinant visfatin induced dose-dependent production of pro-inflammatory IL-1β, TNF-α, and IL-6 as well as anti-inflammatory cytokines like IL-10, and IL-1 receptor antagonist in human monocytes. *In vivo* intraperitoneal injections of recombinant murine visfatin significantly increased circulating IL-6 levels and IL-6 mRNA expression in the small intestine in mice [150]. Other studies demonstrated that visfatin was also synthesized and released by neutrophils in response to inflammatory stimuli and that it functioned as an inhibitor of apoptosis resulting from variety of inflammatory stimuli. Visfatin was expressed at high levels in neutrophils harvested from septic critically ill patients and contributed to prolonged neutrophil survival in clinical sepsis [151]. The physiology of visfatin revealed that it is upregulated in the acute lung injury and sepsis as well as in inflammatory bowel disease [152].

**Figure 3:**
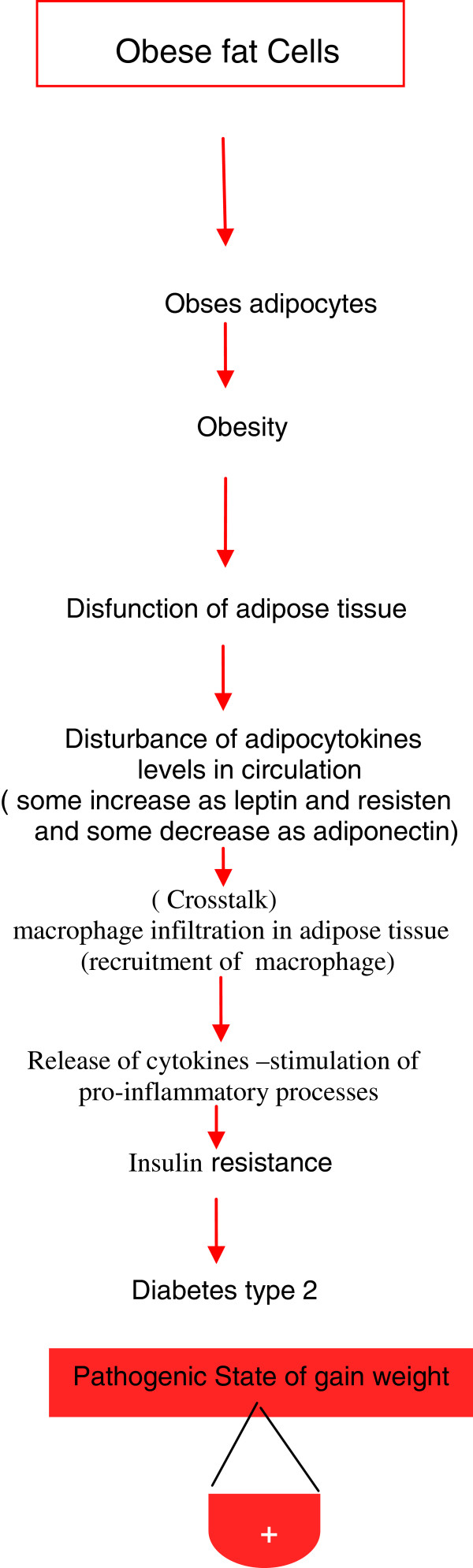
Roles of adipose tissues and adipocytokines during obesity progression and initiation of inflammation.

General physiological functions of leptin and other adipocytokines are important in mediating the physiological role of adipose tissue in different models. Either adipocytes or immune cells and their secretory and metabolic activities should be taken under consideration (Figures 3) [11]. Therefore, immunotherapy deserves to be considered as a promising approach to treat the endocrino-metabolic disorders associated with excessive fat mass development.

Important advances in our understanding of the relationships among adipokinesmetabolism and the immune response have been clarified in the past 10 years. White adipose tissue has served as a highly active organ that releases a plethora of immune and inflammatory mediators that are involved in many diseases [153].

### Future prospects

Several *in vitro* and *in vivo* experiments have confirmed that adipokines have numerous important functions in the body. Research in this area is increasing and tremendous efforts have been made in exploring the physiological mechanisms of adipokines action in metabolic disorders andinflammatory autoimmune disorders. The exact mechanisms and the roles of adipokines remain hazy, and future research will further underpin the potential prospects for therapeutic action.

Much compelling evidence has shown the promotion of inflammation by elevating levels of leptin and resistin. It is conceivable that control of circulating levels of leptin and resistin might prevent inflammatory diseases. Leptin receptor could be stopped from activation with antibodies or legend This means the energy intake role of leptin should not be perturbed because development of hyperphagia and obesity might occur. At present, different studies are underway that are designed to gain insights into the known adipokines, their genetic bases and the cellular events that are taking place in the promotion of inflammatory ailments through the modulation of the adipokines. However, many questions need to be addressed before adipokines can be used as therapeutic targets in inflammatory complications. The depth of the mechanism and the signaling pathways of adipokines presented here are still incomplete and need future attention to elaborate the specific role of each adipokine. Nevertheless, studies that are able to clarify the role of adipokines in the different models, may demonstrate that these adipokines can indeed be essential targets for pharmacotherapeutic agents for the treatment of obesity-induced inflammatory diseases.

## Conclusions

Physiological functions of adipocytokines and cytokines have principle roles in different styles and tissues. Adipokines (leptin, resistin and visfatin) could serve as a missing link in the causal relationship between psoriasis and comorbidities and may provide a biomarker for disease severity such as obesity and diabetes, risk of comorbidities and treatment success. Additionally, adipocytokines have numerous anti-inflammatory actions. Visfatin also mimics insulin effects. The functional balance of adipocytes and immune cells that is needed for them to exert their metabolic activities should be taken under consideration. Development of novel therapeutic procedures for obesity and obesity-associated diseases possibly could be achieved through an integral insight into leptin, resistin and visfatin, as well as insight into other adipokine functions and their links to inflammation.

## Abbreviations

AMPK: AMP-activated protein kinase; ARC: Arcuate nucleus; BAT: Brown adipose tissue; BMI: Body mass index; CNS: Central nervous system; COX-2: Cyclo-oxygenase 2; DMH: Dorsomedial nucleus; GSK-3: Glycogen synthase kinase-3; IL-6: Interleukin-6; iNOS: Inducible nitric oxide synthase; IRS: Insulin receptor substrate; LHA: Lateral hypothalamic area; LPS: Lipopolysaccharides; MAPK: Mitogen-activated protein kinase; MCP-1: Monocyte chemoattractant protein-1; NF-κB: Nuclear factor-kappa B; PMBCs: Peripheral blood mononuclear cells; PMV: Ventral premamillary nucleus; PVH: Paraventricular nucleus; RELM: Resistin-like molecule; TH: T-helper; TNFα: Tumor necrosis factor α; TRL: Triglyceride-rich lipoproteins; VEGF: Vascular endothelial growth factor; VMH: Ventromedial nucleus; WAT: White adipose tissue

## Competing interests

The authors declare no competing interests.

## Authors’ contributions

EAA-S designed, drafted the manuscript and draw the figures. AS added new sections, rewrite another and reviewed the article, In general the work is supported by the equal contribution of the authors. Both authors read and approved the final manuscript.
